# Supplementary material for: Tracing the origins of Plasmodium vivax resurgence after malaria elimination on Aneityum Island in Vanuatu
Source: Commun Med (Lond). 2024 May 18;4:91. doi: 10.1038/s43856-024-00524-9 (PMC11102431; doi:10.1038/s43856-024-00524-9)
Supplement: Supplementary file 2 — Description of Additional Supplementary Files [file 43856_2024_524_MOESM2_ESM.pdf]

Description of Additional Supplementary Files

File name: Supplementary Data 1
Description: PCR primer sequences

File name: Supplementary Data 2
Description: Numbers of samples successfully amplified (NS) and alleles detected (NA) for each microsatellite marker

File name: Supplementary Data 3
Description: Frequencies of microsatellite haplotypes detected in this study

File name: Supplementary Data 4
Description: Primary alleles detected at 12 microsatellite loci
