# Supplementary material for: Tracing the origins of Plasmodium vivax resurgence after malaria elimination on Aneityum Island in Vanuatu
Source: Commun Med (Lond). 2024 May 18;4:91. doi: 10.1038/s43856-024-00524-9 (PMC11102431; doi:10.1038/s43856-024-00524-9)
Supplement: Supplementary file 7 — Reporting Summary [file 43856_2024_524_MOESM7_ESM.pdf]

nature portfolio

Corresponding author(s): Akira Kaneko

Last updated by author(s): Apr 30, 2024

Reporting Summary

Nature Portfolio wishes to improve the reproducibility of the work that we publish. This form provides structure for consistency and transparency in reporting. For further information on Nature Portfolio policies, see our Editorial Policies and the Editorial Policy Checklist.

Statistics

For all statistical analyses, confirm that the following items are present in the figure legend, table legend, main text, or Methods section.

n/a	Confirmed	
☐	☒	The exact sample size (*n*) for each experimental group/condition, given as a discrete number and unit of measurement
☐	☒	A statement on whether measurements were taken from distinct samples or whether the same sample was measured repeatedly
☒	☐	The statistical test(s) used AND whether they are one- or two-sided<br>*Only common tests should be described solely by name; describe more complex techniques in the Methods section.*
☐	☒	A description of all covariates tested
☒	☐	A description of any assumptions or corrections, such as tests of normality and adjustment for multiple comparisons
☐	☒	A full description of the statistical parameters including central tendency (e.g. means) or other basic estimates (e.g. regression coefficient) AND variation (e.g. standard deviation) or associated estimates of uncertainty (e.g. confidence intervals)
☒	☐	For null hypothesis testing, the test statistic (e.g. *F*, *t*, *r*) with confidence intervals, effect sizes, degrees of freedom and *P* value noted<br>*Give P values as exact values whenever suitable.*
☐	☒	For Bayesian analysis, information on the choice of priors and Markov chain Monte Carlo settings
☐	☒	For hierarchical and complex designs, identification of the appropriate level for tests and full reporting of outcomes
☒	☐	Estimates of effect sizes (e.g. Cohen's *d*, Pearson's *r*), indicating how they were calculated

Our web collection on statistics for biologists contains articles on many of the points above.

Software and code

Policy information about availability of computer code

Data collection	Peak Scaneer 2 (Thermo Scientific)
Data analysis	Arlequin ver. 3.5.2.2, LIAN ver. 3.7, Populations ver. 1.2.32, MEGA ver.11, STRUCTURE ver. 2.3.4, STRUCTURE HARVESTER Web ver. 0.6.94, R ver. 4.2.1

For manuscripts utilizing custom algorithms or software that are central to the research but not yet described in published literature, software must be made available to editors and reviewers. We strongly encourage code deposition in a community repository (e.g. GitHub). See the Nature Portfolio guidelines for submitting code & software for further information.

Data

Policy information about availability of data

All manuscripts must include a data availability statement. This statement should provide the following information, where applicable:

- Accession codes, unique identifiers, or web links for publicly available datasets
- A description of any restrictions on data availability
- For clinical datasets or third party data, please ensure that the statement adheres to our policy

All data are available from the authors upon request.

Human research participants

Policy information about studies involving human research participants and Sex and Gender in Research.

Reporting on sex and gender	In this study, only the word "sex" is used.<br>Data were collected on the basis of self-reports.
Population characteristics	Vanuatu people aged 0-70 years participated in the study.<br>All participants in the study had taken blood samples to test for Plasmodium infection.<br>All inhabitants of Aneityum Island were given anti-malarial drugs in 1991.
Recruitment	All inhabitants of the islands of Aneityum and Tanna were included.<br>The 2002 church meeting covered all visitors to Aneityum Island from outside.
Ethics oversight	Tokyo Women's Medical University and the Ministry of Health in Vanuatu

Note that full information on the approval of the study protocol must also be provided in the manuscript.

Field-specific reporting

Please select the one below that is the best fit for your research. If you are not sure, read the appropriate sections before making your selection.

☒ Life sciences ☐ Behavioural & social sciences ☐ Ecological, evolutionary & environmental sciences

For a reference copy of the document with all sections, see nature.com/documents/nr-reporting-summary-flat.pdf

Life sciences study design

All studies must disclose on these points even when the disclosure is negative.

Sample size	No calculation of the required sample size has been carried out.<br>As the samples were obtained from all islanders living in the target area. So, the sample size is considered sufficient
Data exclusions	Samples that were negative for P. vivax by PCR, those that were positive but had low DNA concentrations or incomplete genotyping were excluded.
Replication	Confirmed
Randomization	The sample was divided according to the year and island from which it was collected.
Blinding	Blinding is not possible because of the groupings by year and island and the sample collection on site each time.

Reporting for specific materials, systems and methods

We require information from authors about some types of materials, experimental systems and methods used in many studies. Here, indicate whether each material, system or method listed is relevant to your study. If you are not sure if a list item applies to your research, read the appropriate section before selecting a response.

Materials & experimental systems

n/a	Involved in the study	
☒	☐	Antibodies
☒	☐	Eukaryotic cell lines
☒	☐	Palaeontology and archaeology
☒	☐	Animals and other organisms
☒	☐	Clinical data
☒	☐	Dual use research of concern

Methods

n/a	Involved in the study	
☒	☐	ChIP-seq
☒	☐	Flow cytometry
☒	☐	MRI-based neuroimaging
